# Dyslipidemia, diabetes and periodontal disease, a cross-sectional study in Rafsanjan, a region in southeast Iran

**DOI:** 10.1186/s12903-023-03262-x

**Published:** 2023-08-10

**Authors:** Fatemeh Ayoobi, Somaye Salari Sedigh, Parvin Khalili, Zeinab Sharifi, Hamid Hakimi, Farimah Sardari, Zahra Jamali

**Affiliations:** 1 Occupational Safety and Health Research Center, NICICO, World Safety Organization and Rafsanjan University of Medical Sciences, Rafsanjan, Iran; 2 Clinical Research Development Unit (CRDU), Moradi Hospital, Rafsanjan University of Medical Sciences, Rafsanjan, Iran; 3 Department of Periodontology, Dental School, Rafsanjan University of Medical Sciences, Rafsanjan, Iran; 4 Department of Epidemiology, School of Public Health, Social Determinants of Health Research Center, Rafsanjan University of Medical Sciences, Rafsanjan, Iran; 5 Department of Pediateric Dentistry, Dental School, Rafsanjan University of Medical Sciences, Rafsanjan, Iran; 6 Immunology of Infectious Diseases Research Center, Rafsanjan University of Medical Sciences, Rafsanjan, Iran; 7 Department of Microbiology, Rafsanjan University of Medical Sciences, Rafsanjan, Iran; 8 Department of Oral Medicine, Dental School, Non-communicable Diseases Research Center, Rafsanjan University of Medical Sciences, Rafsanjan, Iran; 9 Non-Communicable Diseases Research Center, Rafsanjan University of Medical Sciences, Rafsanjan, Iran; 10 Clinical Research Development Unit (CRDU), Niknafs Hospital, Rafsanjan University of Medical Sciences, Rafsanjan, Iran

**Keywords:** Dyslipidemia, Diabetes, Periodontal disease, Prospective Epidemiological Research Studies in IrAN (PERSIAN)

## Abstract

**The objectives:**

The association between dyslipidemia, diabetes and alterations in periodontal health are inconsistent. The aim of this study was to determine the association between dyslipidemia, diabetes and periodontal disease in the Oral Health Branch of Rafsanjan Cohort Study (OHBRCS).

**Methods:**

Rafsanjan Cohort Study (RCS) was launched in 2015 in Rafsanjan City a region in the southeast of Iran. A total of 8682 participants aged 35–70 years of both gender were recruited into the OHBRCS as a part of RCS. Bleeding on probing (BOP), probing pocket depth (PPD) and Clinical attachment loss (CAL) were used to assess periodontal health status. When CAL progression was ≥ 1 mm and PPD was > 3 mm, it was defined as periodontitis.

**Results:**

The final sample consisted of 6751 individuals with mean age of 47.67 ± 8.79 years. Among this population, 73.32% (n = 4949), 13.75% (n = 928), 59.67% (n = 4028) and 11.76% (n = 794) had BOP, PPD > 3 mm, CAL ≥ 1 mm and periodontitis respectively. The odds of CAL ≥ 1 mm increased 14% in subjects with high LDL cholesterol (OR: 1.14; 95% CI: 1.01–1.30), 17% in subjects with diabetes (OR: 1.17; 95% CI: 1.01–1.36) and 23% in subjects with both dyslipidemia and diabetes (OR: 1.23; 95% CI: 1.05–1.44). Also, the odds of PPD > 3 mm in the group with high total cholesterol (TC) was 16% higher compared to those with normal TC (OR: 1.16; 95% CI: 1.01–1.34).

**Conclusions:**

There was an increased odds in periodontal disease in association with high TC, high LDL cholesterol, diabetes and having both dyslipidemia and diabetes. This suggests that high TC, high LDL cholesterol, diabetes and having both dyslipidemia and diabetes might be potential indicators for the presence of periodontal disease.

## Introduction

Gingivitis is an inflammatory process confined to the gingival caused by microorganisms that accumulate on the tooth surface. If gingivitis left untreated, may develop to periodontitis, which is diagnosed by destruction of tooth supporting structures, eventually leading to tooth mobility and loss [1]. An epidemiological study found that among adults, the prevalence of total periodontitis (including mild, moderate and severe forms of periodontitis) was 47.7% and the percentage of severe periodontitis was 8.5% [2, 3]. According to previous reports, 80% of the population of the United States and 51% of the UK population will develop periodontitis in their lifetime [4]. Also, the prevalence of periodontitis in Iranian adolescents and adults was reported to be 30% and 53% respectively [5]. Periodontitis is caused by a large number of bacterial biofilm, but the immune response of the host can affect the progression of this disease [6]. Thus, systemic diseases affecting host response may be risk factors for periodontitis [7].

Diabetes is a metabolic disease characterized by hyperglycemia which caused by the defects in insulin action and/or secretion [8]. Periodontal disease is considered the sixth complication of diabetes [9]. Evidence suggests that diabetes can initiate or promote the periodontal disease [10]. A previous study reported a relationship between increased severity of periodontitis and type 2 diabetes mellitus [11]. Also, another study showed that 83% of people with diabetes suffer from periodontitis [12]. On the contrary, in study of Ueno et al. no significant association was found between periodontitis and diabetes [13].

Dyslipidemia, which is one of the concerns of modern societies, has known as one of the most important risk factors for cardiovascular diseases. According to recent researches, the prevalence of dyslipidemia was 73–83% among Iranian adults [14, 15]. Recent studies indicated an association between dyslipidemia and alterations in periodontal health [16]. Fentoglu and colleagues concluded that patients with hyperlipidemia manifested higher levels of periodontal parameters compared to healthy individuals [16]. However, other studies not found this relationship [11, 17, 18]. These associations may differ based on some risk factors including race, dietary habits, and lifestyle. So, more studies are necessary to conclude definitive results.

Considering the high prevalence of dyslipidemia and diabetes in Iran [19, 20], the likely effects of these two risk factors on the progression of periodontal disease and the discrepancy of results between different studies, this study was conducted to determine the association between dyslipidemia, diabetes and periodontal disease with a larger sample size in the population of OHBRCS.

## Materials and methods

### Study design and data collection

Rafsanjan Cohort Study (RCS) included in the Prospective Epidemiological Research Studies in IrAN (PERSIAN) [21] was launched in 2015 in the Rafsanjan, a region in the southeast of Iran. This study was designed to recruit a total of 10,000 participants of both genders aged 35–70 years. All participants signed the informed written consent letter [22].

Selected individuals were interviewed using a standardized and detailed questionnaire validated in the PERSIAN [21]. The questionnaires contain demographic data, socioeconomic status, medication and medical history, life style and anthropometric measurements. Alcohol consumption, smoking and opium usage were self-reported. Smoking was defined as having smoked more than 100 cigarettes in lifetime. Alcohol drinker considered as someone who had consumed 200 ml of beer or 45 ml of liquor, once a week for at least six months during his/her lifetime [21]. A participant was defined as opium user if he/she reported consumption of opium for at least once per week for 6 months during his/her lifetime [23]. Physical activity was assessed based on a 22- item questionnaire and the daily physical activity. Wealth score index (WSI) was used to determine Socio-Economic Status (SES) of individuals.

Oral Health Branch of Rafsanjan Cohort Study (OHBRCS) as part of the RCS was established aiming to investigate the most important aspects of dental and oral health of the participants. All recruited individuals of the adult RCS were also invited to participate in the OHBRCS. Finally, a total of 8682 subjects of the adult cohort study entered the OHBRCS. The questionnaires included factors related to oral health and a full-mouth examination were undertaken by trained oral health professionals. A trained assistant who accompanied the dental specialists recorded the data. Three oral medicine specialist, one periodontist and one general dentist were trained and calibrated with each other and everyone with himself/herself during 2 training sessions for proper diagnosis of oral and periodontal diseases. Instruments were unified for all examiners and included dental mirror and dental probe. The periodontal status was determined by clinical attachment loss (CAL), pocket depth (PPD), and bleeding on probing (BOP). Walking probing method by the Williams Probe (Willliams coded, Hu-Friedy, USA, Michigan ”o” probe) was used for measurements. All permanent, fully erupted teeth except all the erupted third were considered for clinical examination. The obtained CAL included the probing depth plus gingival recession.

This study was approved by the Ethics Committee of Rafsanjan University of Medical Sciences (Ethical codes: ID: IR.RUMS.REC.1399.197). In addition, this study was performed in accordance with the guidelines for the report of observational studies in epidemiology (STROBE).

### Definition of terms

Fasting blood glucose (FBG), total cholesterol (TC), low-density lipoprotein (LDL) cholesterol, high-density lipoprotein (HDL) cholesterol and triglycerides (TG) were measured using a biotecnica analyzer (BT 1500, Italy) at the laboratory of Cohort center. Diabetes was described as FBS ≥ 126 mg/dL or receiving the antidiabetic drugs [24]. According to the Third Report of the National Cholesterol Education Program (NCEP-Adult Treatment Panel III), dyslipidemia was defined as LDL ≥ 130 mg/dL, or TC ≥ 200 mg/dL, or HDL ≤ 40 mg/dL in men, and 50 mg/dl in women or TG ≥ 150 mg/dL and or using lipid-lowering medications [25]. High LDL and high TC were considered as LDL ≥ 130 mg/dL or use of lipid-lowering medications for high LDL and TC ≥ 200 mg/dL or use of lipid-lowering medications for high TC. Furthermore, TG ≥ 150 mg/dL or use of lipid-lowering medications for high TG and HDL ≤ 40 mg/dL in men, and 50 mg/dl in women were defined as high TG and low HDL respectively.

Periodontal health indexes were determined in a population with at least 2 teeth (n: 6751). The thresholds for Pocket and CAL were > 3 mm and ≥ 1 mm respectively [26]. When CAL progression was ≥ 1 mm and PPD was > 3 mm, it was defined as periodontitis [27]. However, observed CAL related to causes unrelated to periodontitis was not considered as CAL such as: (1) gingival recession with traumatic origin; (2) dental caries extending in the cervical area of the tooth; (3) the presence of CAL on the distal aspect of a second molar and associated with malposition or extraction of a third molar, (4) an endodontic lesion draining through the marginal periodontium; and (5) the occurrence of vertical root fracture.

### Statistical analyses

Frequency (%) for categorical variables and mean (SD: standard deviation) for the quantitative variables were used and baseline characteristics were compared across the groups of our study using chi-square (χ²) and t-test for categorical and continuous variables, respectively. In addition, we used univariate and multivariate dichotomous logistics regression analysis to determine the odds ratios (ORs) and the corresponding 95% confidence intervals (CI) for the relation of dyslipidemia and diabetes with BOP, PPD, CAL and periodontitis. We used two models in the regression analysis. Potential confounding variables were sequentially entered into multivariate model according to their hypothesized strengths of association with dyslipidemia, diabetes and periodontal health indexes. Variables with a p-value < 0.25 were selected as confounders. Univariate model (crude model) has been stratified on the condition of dyslipidemia and diabetes. Moreover, multivariate adjusted model is adjusted for confounding variables including age (continuous variable), gender (male/ female), education years (continuous variable) and wealth status index, cigarette smoking, opium using, alcohol drinking, body mass index (continuous variable), physical activity level (continuous variable), hypertension (yes/no), CVD history (yes/no), and brushing frequency (categorical variable). As periodontitis prevalence increases significantly after the age of 40 years, this age was selected as cutoff for subgroup analysis [28, 29]. Also, a sub-analysis by category of the number of teeth was done. All analyses were performed through State V.14. All p-values are two-sided.

## Results

The final sample consisted of 6751 individuals, both gender with mean age ± standard deviation (SD) 47.67 ± 8.79 years that 44.07% were men and 55.93% were women. Table 1 compares sociodemographic characteristics, general health status, habits and laboratory tests of the study population between periodontal health indexes (BOP, PPD, CAL and periodontitis). Of the 6751 study patients, 73.32% (n = 4949), 13.75% (n = 928), 59.67% (n = 4028) and 11.76% (n = 794) were diagnosed as presenting with BOP, PPD > 3 mm, CAL ≥ 1 mm and periodontitis respectively. Among this population, the prevalence of high TC, high LDL, low HDL and high TG was 52.07%, 31.15%, 11.54% and 47.42% respectively. Additionally, 19.84% and 72.22% of the study patients diagnosed having diabetes and dyslipidemia respectively. A significant difference was found between periodontal status and physical activity, sociodemographic characteristics, general health status, habits and laboratory tests. Among participants with BOP, PPD > 3 mm, CAL ≥ 1 mm and periodontitis, 18.64%, 26.29%, 21.95% and 28.22% were smokers, respectively. Also, among those who had BOP, PPD > 3 mm, CAL ≥ 1 mm and periodontitis 16.32%, 24.32%, 19.45% and 25.54% were opium user. The frequency of BOP, pocket, CAL and periodontitis was higher in people who brushed more than once a day than in other people and showed a significant difference (p < 0.001).

**Table 1:** Characteristics of the study population, overall and according to the periodontal health indexes (n: 6751).

Characteristics		Overall (n = 6751)	BOP (n = 4949)	P-Value	PPD > 3 mm (n = 928)	P-Value	CAL ≥ 1 mm (n = 4028)	P-Value	Periodontitis (n = 794)	P-Value
**Age- year- no (%)**				< 0.001		0.011		< 0.001		< 0.001
35–45		3083(45.68)	2341(47.30)		382(41.16)		1459(36.22)		301(37.91)	
46–55		2191(32.46)	1604(32.41)		331(35.67)		1501(37.26)		293(36.90)	
≥ 56		1475(21.86)	1004(20.29)		215(23.17)		1068(26.51)		200(25.19)	
Mean ± SD		47.67 ± 8.79	47.32 ± 8.64	< 0.001	48.58 ± 8.46	< 0.001	49.40 ± 8.60	< 0.001	49.20 ± 8.38	< 0.001
**Gender- no (%)**				0.858		< 0.001		< 0.001		< 0.001
Female		3775(55.93)	2771(55.99)		433(46.66)		2059(51.12)		357(44.96)	
Male		2975(44.07)	2178(44.01)		495(53.34)		1969(48.88)		437(55.04)	
**Education**				0.756		< 0.001		< 0.001		< 0.001
Mean ± SD		9.53 ± 4.78	9.54 ± 4.76		8.75 ± 4.72		9.09 ± 4.87		8.52 ± 4.67	
**Physical activity**				0.003		< 0.001		0.011		< 0.001
Mean ± SD		38.85 ± 6.17	38.93 ± 6.31		39.60 ± 6.96		39.01 ± 6.39		39.81 ± 7.19	
**BMI**				0.098		0.009		0.337		< 0.001
Mean ± SD		28.10 ± 4.79	28.16 ± 4.80		27.71 ± 4.74		28.05 ± 4.76		27.54 ± 4.80	
**Wealth score index**				0.929		< 0.001		< 0.001		< 0.001
Mean ± SD		0.16 ± 0.96	0.16 ± 0.95		0.05 ± 0.97		0.12 ± 0.97		0.028 ± 0.968	
**Alcohol consumption- no (%)**				0.128		0.074		0.522		0.080
Yes		638(9.51)	478(9.73)		102(11.12)		388(9.70)		88(11.24)	
No		6068(90.49)	4437(90.27)		815(88.88)		3611(90.30)		695(88.76)	
**Cigarette smoking-no (%)**				0.001		< 0.001		< 0.001		< 0.001
Yes		1323(19.61)	922(18.64)		244(26.29)		884(21.95)		221(28.22)	
No		5424(80.39)	4025(81.36)		684(73.71)		3143(78.05)		562(71.77)	
**Opium consumption- no (%)**				< 0.001		< 0.001		< 0.001		< 0.001
Yes		1171(17.46)	802(16.32)		223(24.32)		778(19.45)		200(25.54)	
No		5535(82.54)	4113(83.68)		694(75.68)		3221(80.55)		583(74.46)	
**Tooth brushing frequency- no (%)**				< 0.001		< 0.001		< 0.001		< 0.001
≤ 1/week		947(14.51)	749(15.55)		185(20.31)		627(16.00)		163(20.95)	
2–6/ week		2107(32.28)	1623(33.69)		341(37.43)		1297(33.10)		299(38.43)	
≥ 1/day		3473(53.21)	2445(50.76)		385(42.26)		1994(50.83)		316(40.62)	
**DBP**				0.002		0.007		< 0.001		0.033
Mean ± SD		70.47 ± 10.24	70.71 ± 10.29		71.32 ± 10.49		71.17 ± 10.12		71.21 ± 10.41	
**SBP**				0.256		< 0.001		< 0.001		< 0.001
Mean ± SD		104.62 ± 16.22	104.76 ± 16.28		106.64 ± 16.60		106.14 ± 16.29		106.57 ± 16.46	
**Hypertension- no (%)**				0.298		0.195		< 0.001		0.045
Yes		1324(19.72)	955(19.41)		197(2141)		893(22.30)		176(22.39)	
No		5390(80.28)	3964(80.59)		723(78.59)		3111 (77.70)		610(77.61)	
**Diabetes- no (%)**				0.01		0.190		< 0.001		0.220
Yes		1327(19.84)	936(19.08)		197(21.44)		887(22.21)		144(18.32)	
No		5363(80.16)	3969(80.92)		722(78.56)		3106(77.79)		642(81.68)	
**Dyslipidemia- no (%)**				0.115		0.338		< 0.001		0.232
Yes		4844(72.22)	3528(71.71)		678(73.54)		2961(73.87)		584(74.02)	
No		1863(27.78)	1392(28.30)		244(26.46)		1047(26.13)		205(25.98)	
**CVD - no (%)**				0.008		0.655		< 0.001		0.465
Yes		487(7.25)	332(6.75)		70(7.61)		339(8.47)		62(7.89)	
No		6227(92.75)	4587(93.25)		850(92.39)		3665(91.53)		724(92.11)	
**TC**				< 0.001		0.192		< 0.001		0.195
High TC- no (%)	3497(52.07)		2499(50.74)		499(54.06)		2162(53.90)		362(45.88)	
Mean ± SD		198.29 ± 37.18	197.82 ± 37.06	0.091	198.63 ± 37.09	0.765	198.96 ± 37.60	0.072	198.96 ± 37.98	0.588
**LDL**				0.076		0.206		< 0.001		0.103
High LDL**-** no **(%)**		2090(31.15)	1503(30.54)		304(32.94)		1360(33.92)		266(33.67)	
Mean ± SD		107.66 ± 29.65	107.55 ± 29.60	0.636	108.84 ± 30.34	0.192	108.10 ± 30.29	0.140	109.02 ± 31.14	0.170
**HDL**				0.889		0.623		0.003		0.806
Low HDL**-** no **(%)**		775(11.54)	570(11.58)		102(11.06)		424(10.58)		89(11.28)	
Mean ± SD		58.14 ± 10.76	58.06 ± 10.61	0.295	57.26 ± 10.86	0.007	57.99 ± 10.71	0.170	58.14 ± 10.76	0.004
**TG**				0.688		0.897		0.004		0.601
High TG**-** no **(%)**		3183(47.42)	2327(47.27)		439(47.61)		1958(48.85)		381(48.29)	
Mean ± SD		167.52 ± 106.61	165.95 ± 103.55	0.045	168.18 ± 92.54	0.839	169.72 ± 102.74	0.040	170.41 ± 95.01	0.417
**FBS**				0.262		0.014		< 0.001		< 0.001
Mean ± SD		110.21 ± 33.98	109.93 ± 33.59		112.78 ± 37.38		112.05 ± 35.08		104.07 ± 38.61	

Abbreviations: BOP: bleeding on probing, CAL: Clinical attachment loss, BMI: Body mass index, DBP; Diastolic blood pressure, (SBP): Systolic blood pressure, CVD: Cardiovascular diseases, FBS: Fast blood sugar, TG: Triglycerides, TC: Total cholesterol,

Table 2 shows the associations of dyslipidemia and diabetes with periodontal health indexes according to age group in multivariate logistic regression models. In the crude model the odds of BOP decreased in subject with high TC (OR: 0.82; 95% CI: 0.73–0.91), diabetes (OR: 0.83; 95% CI: 0.73–0.95) and in subject with both dyslipidemia and diabetes (OR: 0.84; 95% CI: 0.73–0.97). The odds of CAL in the crude model increased in subjects with dyslipidemia (OR: 1.22; 95% CI: 1.10–1.36), high TC (OR: 1.20; 95% CI: 1.09–1.32), high LDL (OR: 1.38; 95% CI: 1.24–1.54), high TG (OR: 1.15; 95% CI: 1.04–1.27), diabetes (OR: 1.45; 95% CI: 1.28–1.64) and in group with both dyslipidemia and diabetes (OR: 1.49; 95% CI: 1.31–1.71). Also, subjects with Low HDL had lower odds of CAL (OR: 0.80; 95% CI: 0.69–0.93). (Table 2).

**Table 2:** Association of dyslipidemia and diabetes with BOP, PPD > 3 mm, CAL ≥ 1 mm and periodontitis in total and for age group (n = 6749).

variable		Crude model	Adjusted model		
		Total (n = 6749)	Total (n = 6749)	35–39 years (n = 1548)	Over 40 years (n = 5201)
		OR (95%Ci)	OR (95%Ci)	OR (95%Ci)	OR (95%Ci)
**BOP**					
**Dyslipidemia**	No	1	1	1	1
	Yes	0.91(0.80–1.02)	0.96(0.84–1.09)	1.02(0.79–1.32)	0.90(0.77–1.05)
**High TC**	No	1	1	1	1
	Yes	**0.82(0.73–0.91)**	0.89(0.79–1.01)	0.89(0.69–1.15)	**0.85(0.74–0.97)**
**High LDL**	No	1	1	1	1
	Yes	0.90(0.80–1.01)	1.01(0.89–1.15)	1.09(0.78–1.53)	0.94(0.82–1.08)
**Low HDL**	No	1	1	1	1
	Yes	1.01(0.85–1.20)	0.99(0.83–1.19)	1.04(0.71–1.50)	1.01(0.82–1.23)
**High TG**	No	1	1	1	1
	Yes	0.98(0.88–1.09)	0.97(0.86–1.09)	0.91(0.70–1.18)	0.99(0.87–1.13)
**Diabetes**	No	1	1	1	1
	Yes	**0.83(0.73–0.95)**	0.90(0.77–1.04)	0.67(0.42–1.07)	0.87(0.74–1.01)
**Dyslipidemia and diabetes**	No	1	1	1	1
	Yes	**0.84(0.73–0.97)**	0.91(0.78–1.06)	0.70(0.41–1.19)	0.88(0.75–1.02)
**Pocket > 3 mm**					
**Dyslipidemia**	No	1	1	1	1
	Yes	1.08(0.92–1.26)	1.15(0.97–1.37)	0.95(0.65–1.93)	1.19(0.98–1.44)
**High TC**	No	1	1	1	1
	Yes	1.10(0.95–1.26)	**1.16(1.01–1.34)**	1.12(0.77–1.62)	1.14(0.97–1.35)
**High LDL**	No	1	1	1	1
	Yes	1.10(0.94–1.27)	1.15(0.98–1.35)	1.38(0.88–2.17)	1.10(0.93–1.31)
**Low HDL**	No	1	1	1	1
	Yes	1.95(0.76–1.18)	1.09(0.87–1.37)	0.96(0.55–1.68)	1.12(0.87–1.44)
**High TG**	No	1	1	1	1
	Yes	1.01(0.88–1.16)	0.97(0.84–1.13)	0.78(0.53–1.14)	1.01(0.86–1.19)
**Diabetes**	No	1	1	1	1
	Yes	1.10(0.93–1.31)	1.03(0.85–1.24)	1.07(0.54–2.11)	1.00(0.82–1.21)
**Dyslipidemia and diabetes**	No	1	1	1	1
	Yes	1.16(0.97–1.39)	1.10(0.91–1.34)	0.93(0.42–2.05)	1.11(0.91–1.36)
**CAL ≥ 1 mm**					
**Dyslipidemia**	No	1	1	1	1
	Yes	**1.22(1.10–1.36)**	1.05(0.93–1.18)	0.93(0.74–1.17)	1.10(0.96–1.27)
**High TC**	No	1	1	1	1
	Yes	**1.20(1.09–1.32)**	0.96(0.86–1.08)	1.08(0.86–1.36)	1.00(0.89–1.13)
**High LDL**	No	1	1	1	1
	Yes	**1.38(1.24–1.54)**	1.09(0.96–1.23)	**1.42(1.05–1.90)**	**1.14(1.01–1.30)**
**Low HDL**	No	1	1	1	1
	Yes	**0.80(0.69–0.93)**	0.98(0.83–1.15)	0.73(0.52–1.03)	0.97(0.80–1.18)
**High TG**	No	1	1	1	1
	Yes	**1.15(1.04–1.27)**	1.03(0.92–1.15)	0.93(0.73–1.17)	1.03(0.91–1.17)
**Diabetes**	No	1	1	1	1
	Yes	1.45(1.28–1.64)	1.01(0.87–1.16)	0.77(0.49–1.22)	**1.17(1.01–1.36)**
**Dyslipidemia and diabetes**	No	1	1	1	1
	Yes	1.49(1.31–1.71)	1.07(0.92–1.25)	0.78(0.47–1.31)	**1.23(1.05–1.44)**
**Periodontitis**					
**Dyslipidemia**	No	1	1	1	1
	Yes	1.11 (0.94–1.31)	1.18(0.98–1.42)	1.12(0.72–1.74)	1.18(0.96–1.44)
**High TC**	No	1	1	1	1
	Yes	1.09(0.94–1.27)	1.13(0.96–1.32)	1.28(0.84–1.96)	1.09(0.92–1.30)
**High LDL**	No	1	1	1	1
	Yes	1.14(0.97–1.33)	1.16(0.97–1.38)	1.59(0.95–2.65)	1.12(0.93–1.34)
**Low HDL**	No	1	1	1	1
	Yes	0.97(0.77–1.23)	1.16(0.91–1.49)	0.89(0.45–1.75)	1.20(0.92–1.57)
**High TG**	No	1	1	1	1
	Yes	1.04(0.90–1.21)	1.02(0.90–1.19)	0.85(0.55–1.33)	1.03(0.87–1.23)
**Diabetes**	No	1	1	1	1
	Yes	1.20(1.01–1.44)	1.05(0.86–1.28)	1.22(0.57–2.61)	1.05(0.86–1.29)
**Dyslipidemia and diabetes**	No	1	1	1	1
	Yes	1.26(0.04–1.52)	1.16(0.94–1.43)	1.40(0.62–3.16)	1.15(0.93–1.42)

Adjusted model is adjusted for confounding variables including age (continuous variable), gender (male/ female), education years (continuous variable) and wealth status index, cigarette smoking, opium using, alcohol drinking, body mass index (continuous variable), physical activity level (continuous variable), hypertension (yes/no), CVD history (yes/no), and brushing frequency (categorical variable).

Abbreviations: BOP: bleeding on probing; CAL: Clinical attachment loss; TG: Triglycerides; TC: Total cholesterol.

In adjusted OR, the association measurement estimated that in participants aged over 40 years, the odds of BOP decreased 15% in individuals with high TC (OR: 0.85; 95% CI: 0.74–0.97) and the odds of CAL increased 14% in subjects with High LDL (OR: 1.14; 95% CI: 1.01–1.30), 17% in subjects with diabetes (OR: 1.17; 95% CI: 1.01–1.36) and 23% in subjects with both dyslipidemia and diabetes (OR: 1.23; 95% CI: 1.05–1.44) (Table 2).

The adjusted results showed that in 35–39 years old group, frequency of CAL in those with high LDL was higher than in participants with normal LDL (OR: 1.42; 95% CI: 1.05–1.90). In total papulation the odds of Pocket in the group with high TC was 16% higher compared to those with normal TC (OR: 1.16; 95% CI: 1.01–1.34) in fully adjusted model (Table 2).

Table 3 shows the associations of dyslipidemia and diabetes with periodontal health indexes according to the number of teeth in multivariate logistic regression models. In fully adjusted model, in participants > 8 teeth, the odds of PPD increased about 31% in individuals with dyslipidemia (OR: 1.31; 95% CI: 1.03–1.66) and 28% in individuals with high TC 1.28 (OR: 1.28; 95% CI: 1.04–1.58).

**Table 3:** Association of dyslipidemia and diabetes with BOP, PPD > 3 mm and CAL ≥ 1 mm and periodontitis based on the number of teeth (n = 6751).

variable		Crude model		Adjusted model	
		≤ 8 teeth (n = 3591)	> 8 teeth(n = 3160)	≤ 8 teeth (n = 3591)	> 8 teeth(n = 3160)
		OR (95%Ci)	OR (95%Ci)	OR (95%Ci)	OR (95%Ci)
**BOP**					
**Dyslipidemia**	No	1	1	1	1
	Yes	0.88(0.75–1.04)	0.94(0.78–1.12)	0.94(0.78–1.12)	0.98(0.80–1.19)
**High TC**	No	1	1	1	1
	Yes	**0.81(0.69–0.94)**	0.84(0.71–0.98)	0.89(0.75–1.03)	0.91(0.76–1.08)
**High LDL**	No	1	1	1	1
	Yes	0.92(0.78–1.09)	0.89(0.75–1.04)	1.06(0.88–1.28)	0.97(0.81–1.16)
**Low HDL**	No	1	1	1	1
	Yes	0.96(0.76–1.22)	1.10(0.84–1.36)	0.99(0.77–1.29)	0.99(0.77–1.28)
**High TG**	No	1	1	1	1
	Yes	0.98(0.84–1.14)	0.98(0.84–1.14)	0.97(0.82–1.14)	0.98(0.82–1.15)
**Diabetes**	No	1	1	1	1
	Yes	0.85(0.70–1.04)	**0.83(0.70–0.99)**	0.91(0.72–1.15)	0.87(0.72–1.06)
**Dyslipidemia and diabetes**	No	1	1	1	1
	Yes	0.84 (0.68–1.04)	0.84(0.70–1.02)	0.92(0.72–1.18)	0.89(0.72–1.09)
**Pocket > 3 mm**					
**Dyslipidemia**	No	1	1	1	1
	Yes	1.00(0.80–1.25)	1.13(0.90–1.42)	1.01(0.79–1.29)	**1.31(1.03–1.66)**
**High TC**	No	1	1	1	1
	Yes	1.00(0.81–1.22)	1.14(0.94–1.39)	1.05(0.84–1.31)	**1.28(1.04–1.58)**
**High LDL**	No	1	1	1	1
	Yes	1.11(0.89–1.39)	1.03(0.85–1.26)	1.19(0.94–1.52)	1.14(0.92–1.43)
**Low HDL**	No	1	1	1	1
	Yes	0.82(0.58–1.16)	1.03(0.77–1.37)	0.89(0.62–1.27)	1.26(0.93–1.70)
**High TG**	No	1	1	1	1
	Yes	0.99(0.81–1.21)	1.02(0.84–1.23)	0.91(0.73–1.14)	1.04(0.85–1.28)
**Diabetes**	No	1	1	1	1
	Yes	1.16(0.88–1.52)	1.01(0.81–1.26)	1.02(0.76–1.39)	1.00(0.78–1.27)
**Dyslipidemia and diabetes**	No	1	1	1	1
	Yes	1.15(0.86–1.53)	1.08(0.86–1.36)	1.04(0.76–1.43)	1.15(0.89–1.47)
**CAL ≥ 1 mm**					
**Dyslipidemia**	No	1	1	1	1
	Yes	**1.29(1.11–1.49)**	1.07(0.90–1.27)	1.06(0.90–1.24)	1.03(0.85–1.24)
**High TC**	No	1	1	1	1
	Yes	**1.20(1.05–1.37)**	1.08(0.93–1.26)	0.98(0.85–1.14)	0.95(0.80–1.13)
**High LDL**	No	1	1	1	1
	Yes	**1.46(1.26–1.70)**	1.15(0.97–1.35)	1.17(0.99–1.38)	1.02 (0.85–1.22)
**Low HDL**	No	1	1	1	1
	Yes	0.81(0.66-1.00)	**0.72(0.57–0.89)**	0.95(0.75–1.19)	0.97(0.76–1.23)
**High TG**	No	1	1	1	1
	Yes	**1.27(1.11–1.45)**	1.00(0.86–1.17)	1.09(0.94–1.26)	0.96(0.82–1.13)
**Diabetes**	No	1	1	1	1
	Yes	**1.52(1.26–1.83)**	1.17(0.98–1.40)	1.13(0.91–1.40)	0.91(0.75–1.12)
**Dyslipidemia and diabetes**	No	1	1	1	1
	Yes	**1.64(1.35–2.01)**	1.13(0.94–1.38)	1.25(0.99–1.57)	0.93(0.76–1.15)
**Periodontitis**					
**Dyslipidemia**	No	1	1	1	1
	Yes	1.09(0.85–1.40)	1.08(0.85–1.35)	1.10(0.83–1.44)	1.26(0.98–1.61)
**High TC**	No	1	1	1	1
	Yes	1.03(0.82–1.29)	1.08(0.88–1.31)	1.06(0.83–1.35)	1.19(0.96–1.48)
**High LDL**	No	1	1	1	1
	Yes	1.15(0.90–1.48)	1.05(0.85–1.29)	1.19(0.91–1.56)	1.16(0.93–1.46)
**Low HDL**	No	1	1	1	1
	Yes	0.81(0.55–1.20)	1.05(0.78–1.42)	0.90(0.60–1.36)	1.33(0.98–1.82)
**High TG**	No	1	1	1	1
	Yes	1.09(0.87–1.37)	0.99(0.81–1.21)	1.00(0.79–1.28)	1.04(0.84–1.28)
**Diabetes**	No	1	1	1	1
	Yes	1.26(0.94–1.70)	1.04(0.83–1.31)	1.03(0.74–1.44)	0.06(0.83–1.37)
**Dyslipidemia and diabetes**	No	1	1	1	1
	No	1.29(0.95–1.76)	1.11(0.88–1.41)	1.10(0.78–1.56)	1.19(0.92–1.55)

Adjusted model is adjusted for confounding variables including age (continuous variable), gender (male/ female), education years (continuous variable) and wealth status index, cigarette smoking, opium using, alcohol drinking, body mass index (continuous variable), physical activity level (continuous variable), hypertension (yes/no), CVD history (yes/no), and brushing frequency (categorical variable).

Abbreviations: BOP: bleeding on probing; CAL: Clinical attachment loss; TG: Triglycerides; TC: Total cholesterol.

## Discussion

The present study is a population-based study aimed to evaluate the prevalence of periodontal health indexes in relation to diabetes and dyslipidemia in the participants of Oral Health Branch of Rafsanjan Cohort Study (OHBRCS). The number of study patients involved in the present study (n = 8682) represents the largest population in an oral health cohort study in Iran. Of the 6751 study participants, 73.32%, 13.75%, 59.67% and 11.76% were diagnosed as presenting with BOP, PPD > 3 mm, CAL ≥ 1 mm and periodontitis respectively. Among this population, the prevalence of high TC, high LDL, low HDL and high TG was 52.07%, 31.15%, 11.54% and 47.42% respectively. Additionally, 19.84% and 72.22% of the study patients diagnosed having diabetes and dyslipidemia respectively. One of the main findings of this study was that there was a direct association between having high TC and increased odds of PPD > 3 mm even after adjusting for potential confounding variables such as those related to demographic, lifestyle, history of CVD, history of hypertension and brushing frequency. Also, the odds of CAL ≥ 1 mm increased in subjects with high LDL cholesterol, diabetes and subjects with both dyslipidemia and diabetes showing that high TC, high LDL, diabetes and having both dyslipidemia and diabetes may be associated with periodontal disease.

Although there are some studies regarding the association between periodontal disease and dyslipidemia, the results are inconsistent. Some studies reported an association between periodontitis with total cholesterol [30, 31] or TG [32] or LDL [31] while other studies reported an association between periodontal disease with both total cholesterol and TG [33–35]. Another study reported that TG and LDL levels were higher and HDL level was lower in the group with periodontitis [36, 37]. On the other hand some studies with relatively small sample sizes of 52 to 261 participants did not find any association [11, 17]. This discrepancy may be due to differences in study design, sample size, characteristics of the participants, methodology, variety in diagnostic criteria for periodontal parameters and various definitions of lipid disorders.

In the present study, we observed a positive relation between high TC and increased odds of Pocket. In accordance with this finding, in a similar large cohort study, a significant positive association was found between periodontal Pockets with total cholesterol and also LDL levels [31]. In another study, Pocket and CAL had significant associations with serum levels of LDL, TG and TC [38].

Our study also showed that high levels of LDL increased the odds of CAL about 42% and 14% in the participants of less than 40 and over 40 years respectively. Therefore, health professionals should emphasize the management of high LDL levels even for young people like those with older ages for preventing periodontal disease. In the study of Fentoglu, TG, TC and LDL levels were significantly and positively associated with Pocket, BOP, and CAL. In that study, HDL-C was significantly, but negatively associated with CAL. After multiple regression analyses, TG level was significantly associated with Pocket and BOP [16]. The study of Fatin et al. showed higher values of BOP, Pocket and CAL in the hyperlipidemia group compared to the control group. There was a positive association between TC, LDL, and the value of CAL. Also, an association between TG and both Pocket and CAL was noticed [39]. In study of Su-Jin et al. low HDL in both age groups (under 40 years and over 40 years groups) and high LDL in the over 40 years group were related with periodontitis [28]. Consistent with our study, Machado et al. [18], Su-Jin et al. [28] and katz et al. [30], did not mention a significant association between periodontal status and TG levels.

Hyperlipidemia has a dysregulatory effect on immune system cells and on wound healing; as a result, it increases the susceptibility to periodontitis and other infections. Hyperlipidemia leads to functional abnormalities in polymorphonuclear leukocytes (PMNs), that have a protective role in the early response to periodontal infection [16]. Hyperlipidemia can increase monocyte differentiation process, which results in a change of macrophage subsets and cytokine release at the wound site, impairing the wound-healing processes [39]. It has been demonstrated that hyperlipidemia leads to hyperactivity of white blood cells (WBC) ,which it turn, leads to increased production of oxygen radicals that had been associated with progressive periodontitis in adults [16].

The relationship between diabetes and periodontal disease is applied in both directions that called bilateral relationship [40]. Previous evidence suggests that the risk of periodontitis in health people is approximately 3 to 4 times lower than in subjects with diabetes [10]. Also, Popławska showed that 83% of people with diabetes suffer from periodontitis, which shows a direct link between the two diseases [12]. According to the results of our study and in consistent with some previous studies [11, 41, 42], the odds of CAL increased in diabetes. While, some studies did not find any association between diabetes mellitus and periodontal disease [13, 43]. Differences in methodological approaches and definitions of diabetes may explain the contradictory findings. In the present study, diabetes and having both diabetes and dyslipidemia were related to CAL among the over 40 years group. Therefore, control of these systemic diseases is important for periodontal disease management particularly among people aged over 40 years.

Studies have shown that diabetes and systemic and inflammatory diseases cause periodontitis in such a way that these diseases promote formation and activation of osteoclasts [44] and poor control of blood sugar leads to increased bone loss [45]. Disruption of the homeostatic balance between the host and bacterial responses causes inflammation near the bone and periodontal ligament [45, 46]. Another postulated mechanism for diabetic effects on periodontal disease is that diabetes-enhanced inflammation and apoptosis specifically affect periodontal tissues and change expression of genes related to periodontal destruction [47]. Since the prevalence of diabetes in the Iranian population is increasing [20], it shows the importance of controlling this disease to prevent periodontal disease among other risk factors.

A large number of participants and population-based research are the major strengths of this study. Extensive data collection for potential confounders (demographic, lifestyle, medical history and etc.) is other strength of this study. However, there are some methodological limitations when interpreting our results. First, because this study had a cross-sectional design, it is difficult to make causal inferences based on its findings. Therefore, a cohort study with follow-up in the future will be helpful to determine the causal association between periodontal disease and these systemic diseases. Diagnostic criteria with FBS alone (not in combination with 2hBG and HbA1c) was another limitation of our study. Finally, the information about the severity levels of periodontal indexes (mild, moderate and severe) was not available in the present study.

## Conclusion

The present study found that there was an increased odds in periodontal disease in association with high TC, high LDL, diabetes and having both dyslipidemia and diabetes. This suggests that high LDL, high TC, diabetes and having both dyslipidemia and diabetes might be potential indicators for the presence of periodontal disease.

## Data Availability

The data is not available publicly. However, upon a reasonable request, the data can be obtained from the corresponding author.
